# T213. THE EFFECT OF ACUTE STRESS ON PARANOID THINKING AND CORTISOL DURING SOCIAL INTERACTION IN HIGH AND LOW SCHIZOTYPES

**DOI:** 10.1093/schbul/sby016.489

**Published:** 2018-04-01

**Authors:** James Gilleen

**Affiliations:** Institute of Psychiatry

## Abstract

**Background:**

Paranoid thinking, a common symptom of psychosis and schizophrenia, manifests as a sense of threat and may also be indexed by a lack of trust. Stress, in turn, exacerbates psychosis and paranoia, and is a well-established risk factor for schizophrenia as well as a component in several models of psychosis. The present study aimed to determine the impact that acute stress has on paranoid thinking during social interaction in vivo in high (HSZ) vs low (LSZ) schizotypy using an iterated social reciprocity game. The main hypothesis was that HSZ would anticipate grerater social threat and paranoia at baseline compared to LSZ, and moreover that experimentally-induced stress would exacerbate those differences, and thus show that stress differentially modulates how HSZ model the intentions of others.

**Methods:**

Matched healthy participants were stratified into HSZ (N=17) and LSZ (N=17) groups and were administered a non-financial, social-reciprocity game against benevolent and malevolent opponents under both stress and no-stress conditions. Stress was manipulated using the MIST (Dedovic et al., 2005) stress paradigm. Cortisol was measured from saliva samples acquired before and after the MIST stress task. Anticipation of threat and trust scores were derived from the social interaction task.

**Results:**

At baseline, cortisol levels were not significantly different between HSZ and LSZ but were significantly raised by the stressor task in HSZ (p<.05) but not in LSZ. Higher cortisol at baseline (pre-stress) predicted greater initial and average anticipation of threat (both r=.5, p<.05) (no-stress) to other players; and lower initial trust ratings of malevolent, but not benevolent, players. The MIST task significantly elevated stress ratings compared to baseline (p<.001) and following stress, greater change in cortisol from baseline to post-MIST was associated with lower trust ratings (r=.51, p<.05). In HSZ greater stressor-related cortisol levels correlated with greater anticipation of threat (r=.58, p<.05) and lower trust levels (r=.57, p<.05) which was not apparent in low schizotypes.

**Discussion:**

Acute stress elevates cortisol and paranoia in HSZ – in the form of greater anticipation of threat, and lower trust compared to LSZ. The study adds evidence to the role of stress in exacerbating schizophrenia-like experiences in those who are sub-threshold for schizophrenia and psychosis.

